# Comparing Clinical, Imaging, and Physiological Correlates of Intestinal Pseudo-Obstruction: Systemic Sclerosis vs Amyloidosis and Paraneoplastic Syndrome

**DOI:** 10.14309/ctg.0000000000000206

**Published:** 2020-08-03

**Authors:** Rahul Pamarthy, Antonio Berumen, Margaret Breen-Lyles, Madhusudan Grover, Ashima Makol

**Affiliations:** 1 Division of Gastroenterology and Hepatology, Mayo Clinic, Rochester, Minnesota, USA;; 2 Division of Rheumatology, Mayo Clinic, Rochester, Minnesota, USA.

## Abstract

**INTRODUCTION:**

Intestinal pseudo-obstruction is characterized by impaired transit and luminal dilation in the absence of mechanical obstruction. Our study aims to describe the clinical, radiographic, and physiological findings in pseudo-obstruction associated with systemic sclerosis (SSc), amyloidosis, and paraneoplastic syndrome.

**METHODS:**

A retrospective cohort of patients evaluated at our institution between January 1, 2008, and August 1, 2018, was assembled. Clinical, imaging, and physiological characteristics were abstracted from electronic medical records.

**RESULTS:**

We identified 100 cases of pseudo-obstruction (55 SSc, 27 amyloidosis, and 18 paraneoplastic). Female population predominance was seen in SSc (71%) vs male population in amyloidosis (74%). Most common symptom was abdominal bloating in all 3 groups. Vomiting was more common in SSc than amyloidosis (73% vs 46%, *P* = 0.02). Diarrhea was more common in amyloidosis and SSc compared with paraneoplastic (81% and 67% vs 28%, *P* < 0.01). Weight loss (>5%) was more common in SSc compared with amyloidosis and paraneoplastic (78% vs 31% and 17%, *P* < 0.0001). Only small bowel dilation was seen in 79%, 40%, and 44% and only large bowel dilation in 2%, 44%, and 44% of patients in SSc, amyloidosis, and paraneoplastic, respectively. Five of 8 SSc patients had myopathic and 3 of 5 paraneoplastic had neuropathic involvement on gastroduodenal manometry.

**DISCUSSION:**

SSc-associated pseudo-obstruction demonstrates female population predominance and presents with vomiting, diarrhea, and weight loss. Amyloidosis-associated pseudo-obstruction shows male population predominance. Small bowel is more commonly involved than large bowel on both imaging and transit studies in SSc. Myopathic involvement was more common in SSc, contrary to neuropathic in paraneoplastic syndrome.

## INTRODUCTION

Intestinal pseudo-obstruction is a rare, debilitating disorder characterized by luminal dilation and impaired transit of contents without any radiologic, surgical, or endoscopic evidence of mechanical obstruction. The symptoms can be acute, chronic, or recurrent resembling an obstructing lesion in the intestinal tract (1,2). Causative factors implicated include metabolic, genetic, trauma/surgery, medications or it can be idiopathic in nature (3,4). Systemic sclerosis (SSc), amyloidosis and paraneoplastic syndromes are systemic conditions frequently associated with intestinal pseudo-obstruction.

SSc is a chronic, progressive, multisystem disorder characterized by autoimmunity, vasculopathy, and widespread fibrosis of the skin and internal organs (5,6). Gastrointestinal (GI) involvement is seen in 70%–90% of SSc patients and associated with significant morbidity and mortality (7,8). SSc can involve gut from oral to aboral end, with the esophagus being most commonly involved (9). Intestinal pseudo-obstruction in SSc is poorly understood and has been attributed to an infiltrative or fibrotic myopathic and/or neuropathic process (1,10).

Amyloidosis results from deposition of insoluble fibrils of abnormal amyloid protein in the extracellular spaces of tissues (11,12). The GI tract is commonly involved with mucosal and muscular infiltration of amyloid (13) and/or through neural involvement that can cause altered motility (12). GI manifestations frequently noted are macroglossia, esophageal dysmotility, gastroesophageal reflux disease (GERD), bleeding, and malabsorption (12,14) and are more common in amyloid light-chain (AL) than in the amyloid A (AA) subtype (14).

Paraneoplastic intestinal pseudo-obstruction results from humoral mediators secreted by tumor cells or because of immune response to tumor antigens (15,16). Paraneoplastic GI dysmotility has been most commonly reported in patients with small-cell lung cancer (SCLC) but has also been observed in association with tumors of the stomach, esophagus, pancreas, breast, and ovaries (15,17). These patients usually have 1 or more types of onconeuronal autoantibodies detectable in their blood, the most common being antineuronal nuclear antibody type 1 (ANNA-1)/anti-Hu (18,19).

The description of intestinal pseudo-obstruction in SSc, amyloidosis, and paraneoplastic syndromes has been limited to single-patient case reports and small case series. In addition, these do not provide comprehensive assessment of the spectrum of symptoms, extent of involvement, and physiological alterations associated with the disease. Our aim is to describe the clinical correlates, imaging findings, and physiological studies (scintigraphic transit and manometry) in a large cohort of intestinal pseudo-obstruction associated with SSc, amyloidosis, and paraneoplastic syndrome evaluated at a single tertiary referral center.

## MATERIALS AND METHODS

### Identification of patients

**Figure 1.**
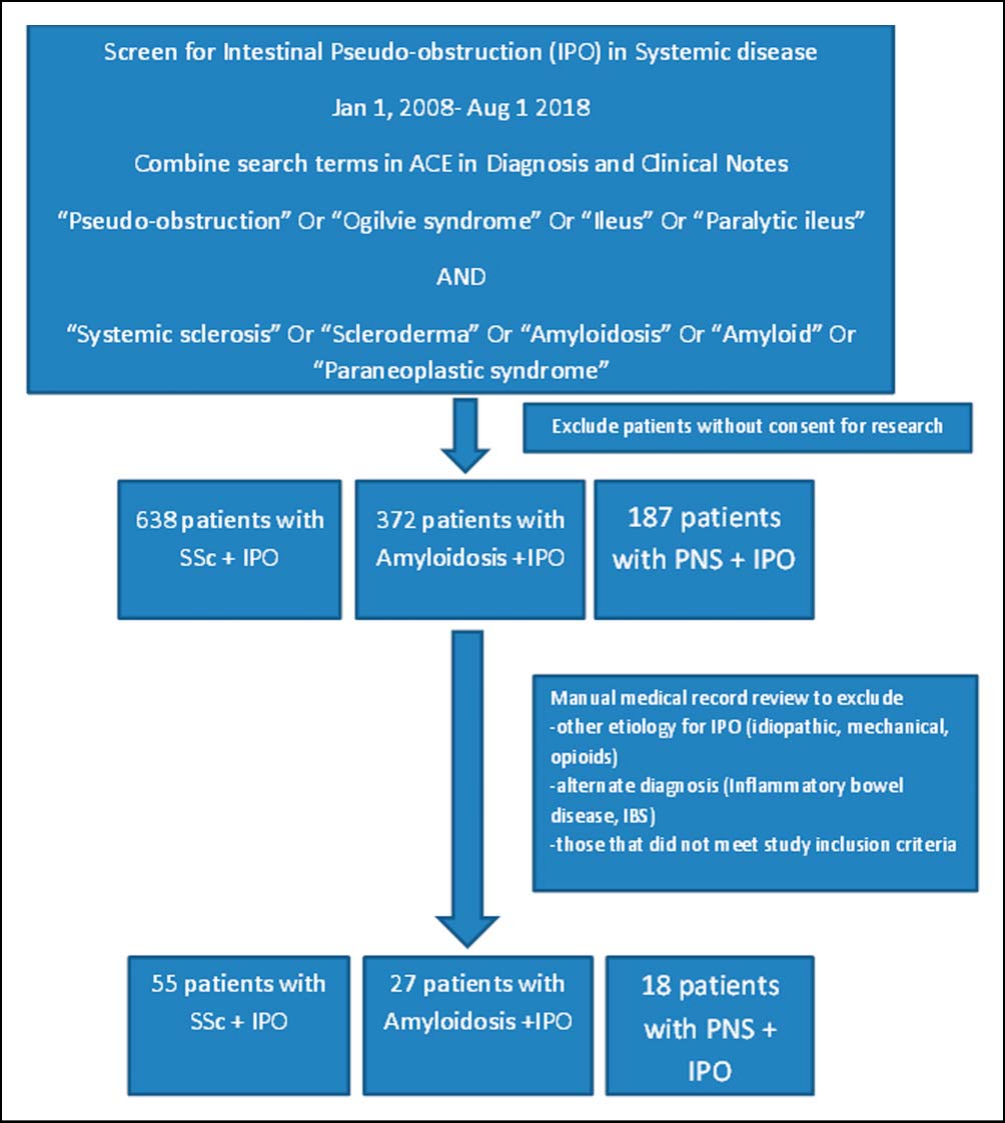
Participant flowchart for identification of pseudo-obstruction in patients with systemic disease.

Patients evaluated for pseudo-obstruction between January 1, 2008, and August 1, 2018, at Mayo Clinic, Rochester, MN, were identified using the text search tool of advanced cohort explorer as the search engine. Both inpatients and outpatients were included. Multiple search terms were used to capture patients with this diagnosis (e.g., pseudo-obstruction, Ogilvie syndrome, ileus, and paralytic ileus) and combined with search terms for those with the 3 systemic diseases of interest (using terms systemic sclerosis, scleroderma, amyloid, amyloidosis, and paraneoplastic syndrome) (Figure 1). All charts were then manually reviewed to confirm that they met inclusion and exclusion criteria as listed further, and those with a confirmed diagnosis of pseudo-obstruction in patients with a physician confirmed clinical diagnosis of SSc, amyloidosis, and paraneoplastic syndrome were included.

### Inclusion criteria

Patients aged 18–80 years with ≥1 symptom(s) suggestive of ileus (abdominal distension, abdominal pain, nausea, vomiting, constipation, and diarrhea) and intestinal dilation (≥2.5 cm in the small intestine, ≥6 cm in the ascending/transverse/descending colon, or ≥8 cm in caecum/sigmoid colon) on abdominal x-ray and/or computed tomography were included. Those with any structural gut lesions on imaging, endoscopy, or laparoscopy were excluded. Both clinical and radiological features needed to be present for inclusion. A physician-confirmed diagnosis of SSc was necessary for inclusion. We also ascertained fulfillment of the ACR/EULAR 2013 classification criteria for SSc. Based on the extent of skin disease, patients with SSc were characterized as limited cutaneous SSc (sclerosis of the skin distal to their elbows and knees), diffuse cutaneous SSc (both proximal and distal sclerosis), or sine scleroderma SSc (typical vascular or internal organ involvement and serological abnormalities in the absence of skin sclerosis). Patients with amyloidosis with histological confirmation of amyloid infiltration in the GI tract or other extraintestinal organs were included. Patients with physician-diagnosed paraneoplastic syndrome were included if they had an active malignancy and/or an onconeural antibody.

### Exclusion criteria

Patients who refused authorization for research using medical chart reviews were excluded. Patients with idiopathic, iatrogenic, endocrine, and metabolic causes of pseudo-obstruction were also excluded.

### Ethics statement

This study was approved by the Institutional Review Board of Mayo Clinic (IRB 18-008132; Approval: September 10, 2018).

### Statistical analysis

Descriptive statistics were used to summarize the demographic characteristics of the cohort. Continuous variables were expressed as means ± SDs and range. Categorical variables were analyzed by χ^2^ test, whereas continuous variables were done by 2-way analysis of variance and Tukey *post hoc* test. *P* values < 0.05 were considered statistically significant for all comparisons. Statistical analysis was performed by using the JMP Pro statistical software (version 14.1.0; SAS Institute, Cary, NC).

## RESULTS

We screened 1,197 medical records manually to identify 100 confirmed cases of intestinal pseudo-obstruction, of which 55 were associated with SSc, 27 with amyloidosis, and 18 with paraneoplastic syndrome (Figure 1).

### Demographics

The demographic characteristics of the cohort are listed in Table 1. Female population (71%) predominance was noted in SSc associated pseudo-obstruction, whereas male population predominance was noted in amyloidosis (74%) and paraneoplastic (56%) groups. White was the most common ethnicity in all 3 groups. The age distribution at disease diagnosis and onset of pseudo-obstruction for the 3 groups is shown in Table 1. SSc patients had a mean interval of 11 years between disease onset and pseudo-obstruction diagnosis; however, these nearly coincided with disease onset in patients with amyloidosis and paraneoplastic syndrome.

**Table 1.**
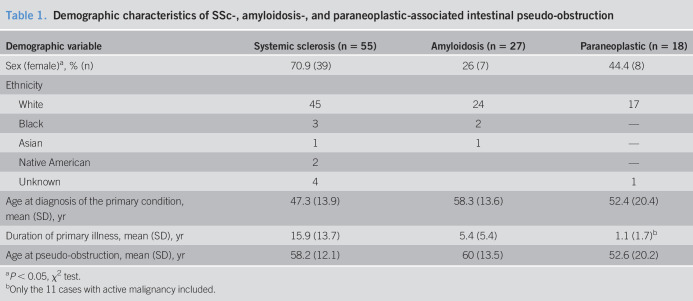
Demographic characteristics of SSc-, amyloidosis-, and paraneoplastic-associated intestinal pseudo-obstruction

### Clinical features of pseudo-obstruction

GI symptoms in SSc, amyloidosis, and paraneoplastic syndrome associated pseudo-obstruction are presented in Table 2. Abdominal bloating was the most common symptom described by >70% patients in all 3 categories. Vomiting was more common in SSc than in amyloidosis (73% vs 46%, *P* = 0.02). Diarrhea was more common in the SSc group than in the paraneoplastic group (67% vs 28%, *P* = 0.003) and in the amyloidosis group when compared with the paraneoplastic group (81% vs 28%, *P* = 0.0003). Weight loss (>5%) was more common in SSc when compared with the amyloidosis (78% vs 31%, *P* < 0.0001) and paraneoplastic syndrome (78% vs 17%, *P* < 0.0001). Malnutrition (body mass index [BMI] < 18.5 kg/m^2^) was seen in 27% of patients with SSc, 17% of patients with paraneoplastic syndrome, and only 8% of patients with amyloidosis.

**Table 2.**
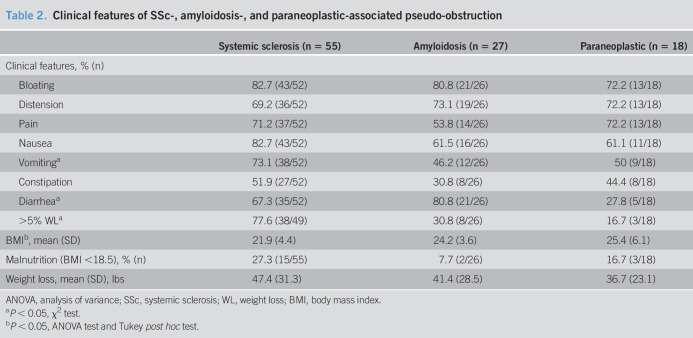
Clinical features of SSc-, amyloidosis-, and paraneoplastic-associated pseudo-obstruction

Among patients with SSc, 45 of 55 (82%) had an upper endoscopy, and 2 of 55 (3.6%) had a breath test for small intestinal bacterial overgrowth (SIBO). Comorbid GI diagnosis in SSc included GERD (73%), esophageal dysmotility (58%), esophagitis (27%), fecal incontinence (18%), esophageal stricture (9%), and gastric antral vascular ectasia (4%). SIBO was diagnosed in 76% (n = 34/45) of patients showing greater than 10^5^ per mL Gram-negative bacilli on the small intestinal aspirate. Comorbid GI features in amyloidosis were GI bleeding (37%), macroglossia (18%), GERD (11%), pelvic floor dysfunction (4%), and fecal incontinence (4%). Comorbid GI features in paraneoplastic syndromes were dysphagia (28%), pelvic floor dysfunction (28%), GERD (11%), and fecal incontinence (6%).

### Features observed in the primary disease

#### Systemic sclerosis

Patients were subclassified into 3 groups based on their skin findings—limited cutaneous SSc (39), diffuse cutaneous SSc (7), and sine scleroderma SSc (9). The features of SSc observed in these patients were Raynaud (96%), sclerodactyly (66%), skin sclerosis proximal to metacarpophalangeal joints (38%), digital ulcers (41%), calcinosis (40%), and telangiectasia (69%). Interstitial lung disease and pulmonary arterial hypertension were seen in 26% and 28%, respectively. Serologically, 87% (n = 40/46), 11% (n = 4/36), 41% (n = 14/34), and 29% (n = 5/17) of patients with SSc had ANNA, anti-Scl-70 antibody, anticentromere antibody, and RNA polymerase III antibody, respectively. Fifty-one of 55 patients met the ACR/EULAR 2013 classification criteria for SSc, whereas the remaining 4 patients had missing data, but they had a well-established clinical diagnosis of SSc made at other institutions. Neurological, cardiac, musculoskeletal, and renal involvements were noted in 2%, 13%, 17%, and 4%, respectively.

#### Amyloidosis

Patient distribution was 22 AL (primary), 4 ATTR (familial), and 1 AA (secondary). Of 22 AL, 11 were kappa light chain, and 10 were lambda light chain restricted type (subtype was not available on 1 patient). Bone marrow biopsies on 21 and abdominal fat pad biopsies on 17 patients showed amyloid deposition. Twenty-two patients (82%) underwent an endoscopic procedure and had amyloid deposition on GI biopsies, but the depth of involvement was available on 15 patients. Of these, 9 had mucosal involvement (3 stomach, 3 duodenum, 1 jejunum, and 2 colon), 10 had submucosal involvement (1 stomach, 5 duodenum, 1 ileum, and 3 colon), 7 had both mucosal and submucosal involvement (2 stomach, 3 duodenum, 1 ileum, and 1 colon), and 2 had transmural involvement (1 ileum and 1 colon). Amyloidosis also involved major organs such as liver (11%), lung (4%), kidney (41%), spleen (7%), nervous system (33%), musculoskeletal (4%), and skin (7%).

#### Paraneoplastic syndrome

Of 18 cases, 11 had an active cancer and 7 had a positive paraneoplastic panel but no detectable cancer. The cancers were ovarian teratoma (n = 1), SCLC (3), both small and squamous cell carcinoma of lung (1), adenocarcinoma of prostate (1), bladder (1), rectal (1), renal (1), neuroendocrine tumor of lung (1), and Hodgkin's lymphoma (1). The antibodies seen in the 7 cases without a detectable cancer were ganglionic acetylcholine receptor (AChR) antibody (n = 3), ANNA-1 (2), voltage-gated potassium channel (VGKC) antibody (1), and VGKC, ANNA-1, and glutamic acid decarboxylase (GAD)-65 (1). The antibody panel was available for 5 patients with cancer: N-methyl d-aspartate receptor antibody (NMDA) (n = 1), ANNA-1 (1), muscle AChR (1), VGKC (1), and collapsing response mediator protein (CRMP)-5, and N-type voltage-gated calcium channel (VGCC) antibody (1). Other paraneoplastic syndromes seen in this cohort of patients were NMDA receptor encephalitis, hypercalcemia, phrenic neuropathy, polycythemia, Cushing syndrome, myelodysplasia, sensory neuropathy, and neurological symptoms. Three patients (17%) had an upper endoscopy, and 1 patient (5%) had breath testing.

### Radiological findings

#### Systemic sclerosis

**Figure 2.**
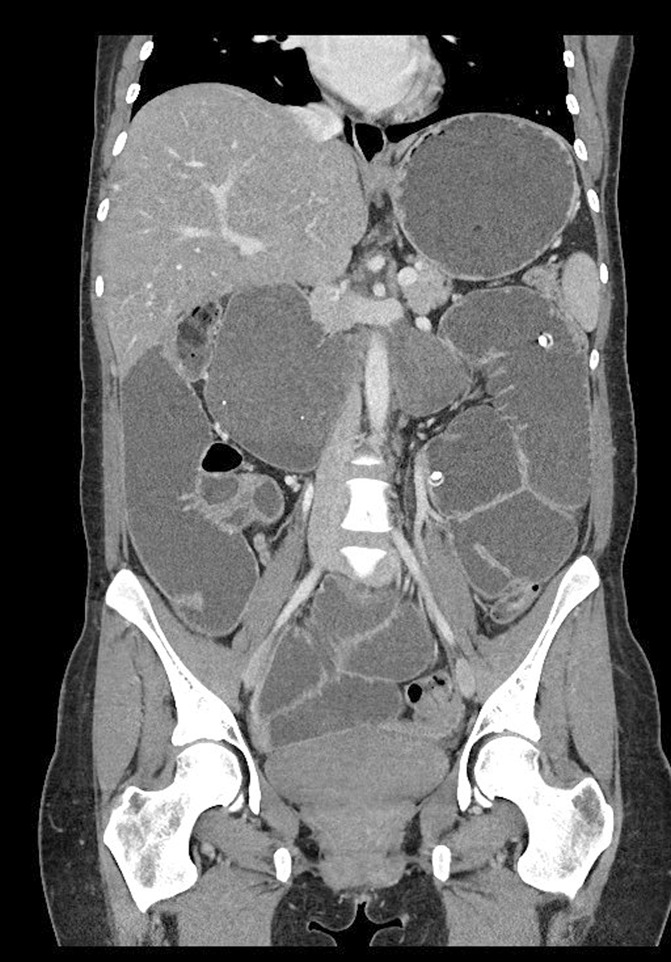
A CT image showing massively dilated small bowel in a patient with SSc. Duodenum dilated up to 8 cm. Features of “hidebound” seen in jejunum. In addition, incidentally seen is a percutaneous jejunostomy tube placed for nutritional support. SSc, systemic sclerosis.

Of the total 47 patients with available imaging, 37 patients had only small bowel dilation, 1 patient had only large bowel, and 9 had both small and large bowel dilations (Table 3). Duodenal dilation was seen in 96%, jejunal in 94% (representation in Figure 2), ileal in 87%, and colonic in 21% of patients.

**Table 3.**
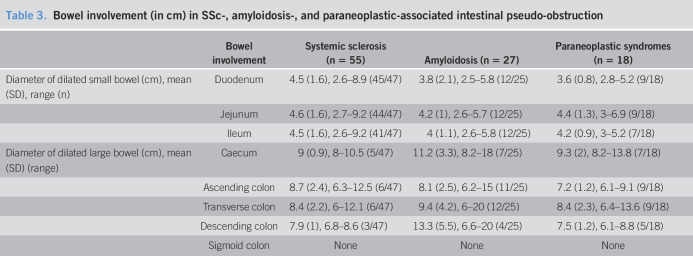
Bowel involvement (in cm) in SSc-, amyloidosis-, and paraneoplastic-associated intestinal pseudo-obstruction

#### Amyloidosis

**Figure 3.**
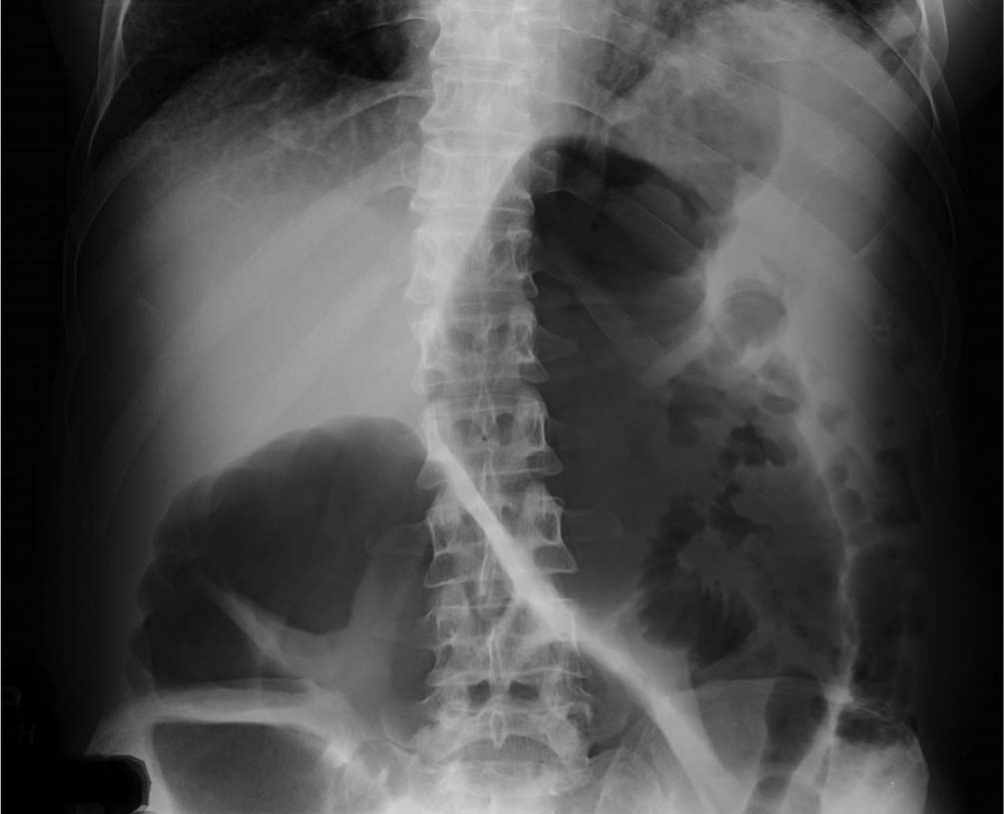
An x-ray showing diffuse marked dilatation of the ascending and transverse colon measuring up to approximately 15 cm in a patient with amyloidosis.

Of the 25 patients with available imaging, 10 had only small bowel dilation, 11 had only large bowel dilation, and 4 had both small and large bowel involved. Duodenal dilation was seen in 48%, jejunal in 48%, ileal in 48%, cecal in 28%, ascending colonic in 44%, transverse colonic in 48% (representation in Figure 3), and descending colonic in 16% of patients.

#### Paraneoplastic syndrome

**Figure 4.**
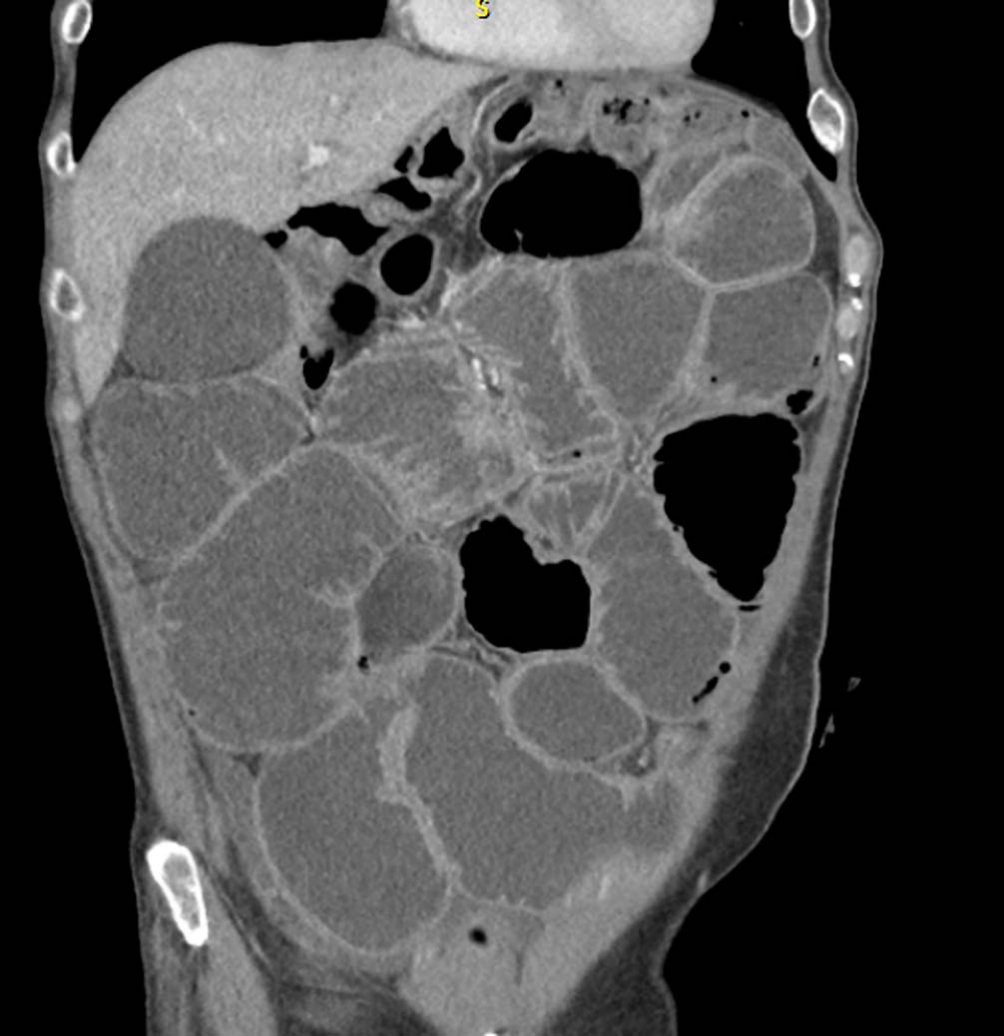
A CT scan showing dilated small bowel without a transition point (suggesting intestinal pseudo-obstruction) in patient with adenocarcinoma of prostate (nonmetastatic).

Of the 18 patients, 8 had only small bowel dilation, 8 had only large bowel and 2 had both small and large bowel involved (representation in Figure 4). Duodenal dilation was seen in 50%, jejunal in 50%, ileal in 39%, cecal in 39%, ascending colonic in 50%, transverse colonic in 50%, and descending colonic in 28% of patients.

### Physiological studies

#### Systemic sclerosis

Gastric emptying (GE) was performed on 28 of 55 (51%) patients with SSc (Table 4). GE at 1 hour was delayed in 8 of 27 (30%) patients and rapid in 5 of 27 (18%) patients. GE at 2 hours was delayed in 15 of 28 (54%) patients and rapid in 6 of 28 (21%) patients. GE at 4 hours was delayed in 15 of 28 (54%) patients and rapid in 4 of 28 (14%) patients. Small bowel emptying was delayed in 17 of 25 (68%) patients. Colon transit was available on 18 patients. Geometric center (GC) at 24 hours was delayed in 9 of 18 (50%) patients, rapid in 2 of 18 (11%) patients, and normal in the rest of the patients. Six patients had GC available at 48 hours, and it was delayed in 4 patients. Eight patients had gastroduodenal manometry, 7 of which were abnormal (5 myopathic process, 1 neuropathic process, and 1 both) (Table 5). Low amplitude of contractions was seen in all the patients at the level of small intestine, and uncoordinated contractility was seen in 2 patients.

**Table 4.**
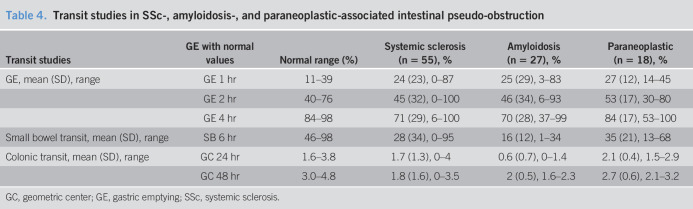
Transit studies in SSc-, amyloidosis-, and paraneoplastic-associated intestinal pseudo-obstruction

**Table 5.**
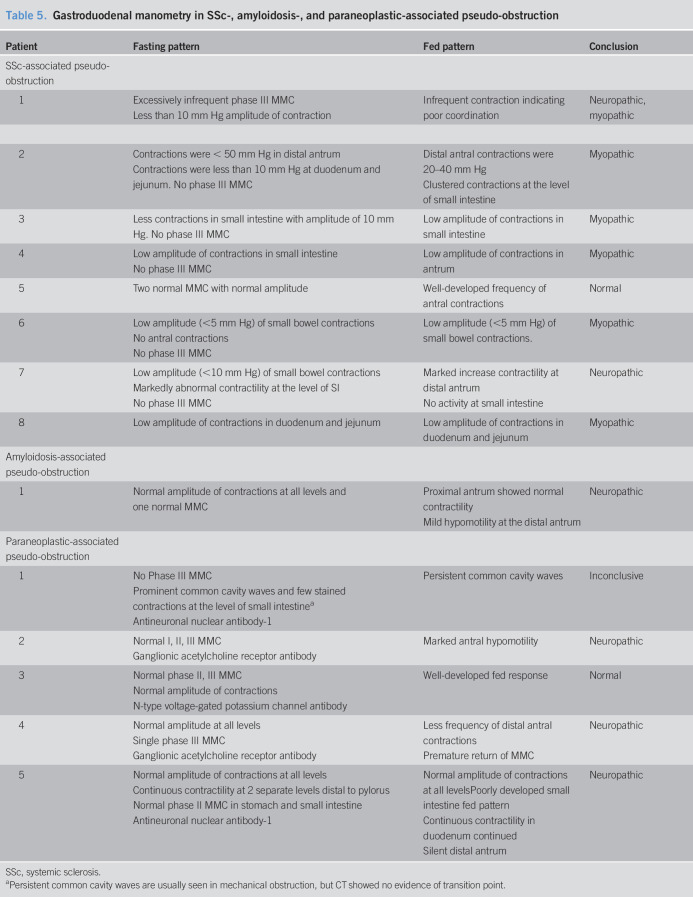
Gastroduodenal manometry in SSc-, amyloidosis-, and paraneoplastic-associated pseudo-obstruction

#### Amyloidosis

Transit was only available in 5 of 27 (18%) patients. GE at 2 hours was delayed in 2 of 5 (40%) patients and rapid in 1 of 5 (20%) patients. GE at 4 hours was delayed in 3 of 5 (60%) patients and rapid in 1 of 5 (20%) patients. Small bowel emptying was delayed in all 5 patients. Colon transit was available on 3 patients, and GC at 24 hours was delayed in all patients. Two of 3 patients had GC available at 48 hours and stayed delayed in both. Only 1 patient had gastroduodenal manometry that was abnormal (neuropathic type). Mild hypomotility at the distal antrum was observed in this patient.

#### Paraneoplastic syndrome

**Figure 5.**
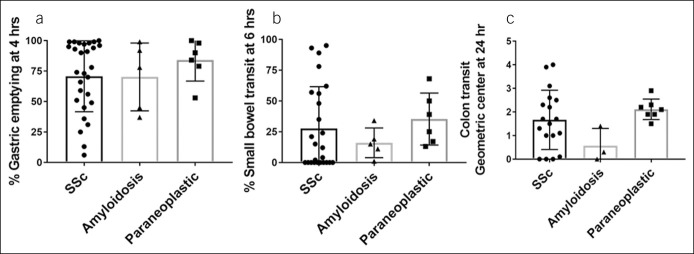
Distribution of gastrointestinal transit parameters in the 3 groups: (**a**) gastric emptying at 4 hours; (**b**) small bowel transit at 6 hours; and (**c**) geometric center for colon transit at 24 hours.

Transit was performed on 6 of 18 (33%) patients. The GE at 2 hours was delayed in 1 of 6 (17%) and rapid in 1 of 6 (17%) patients. The GE at 4 hours was delayed in 2 of 6 (33%) patients and rapid in 1 of 6 (17%) patients. The small bowel emptying was delayed in 4 of 6 (67%) patients. Colon transit was available on 7 patients. GC at 24 hours was delayed in 1 of 7 (14%) patients, and normal in the rest. Five patients had gastroduodenal manometry, 4 of which were abnormal (3 neuropathic process and 1 inconclusive). All patients had good amplitude contractions, but 3 patients had uncoordinated contractions. Figure 5 displays distribution of key transit parameters for the 3 groups.

### Treatment and outcomes

The mean duration of follow-up was similar between the 3 groups (Table 6). Total parenteral nutrition was used for 22%–35% of the patients (SSc > amyloidosis or paraneoplastic). Prokinetic and octreotide use was similar between the 3 groups, whereas antibiotic use for SIBO was significantly higher in SSc group than that in amyloidosis and paraneoplastic groups. Intravenous immunoglobulin (IVIG) and intravenous corticosteroid use for pseudo-obstruction was significantly higher in the paraneoplastic group. All 3 patients with paraneoplastic syndrome receiving corticosteroids for pseudo-obstruction reported improvement in GI symptoms. Of the 4 patients with paraneoplastic syndrome on IVIG, 1 reported no change, 1 worsening of abdominal pain, and 2 reported improvement. However, 1 of those 2 was also concomitantly treated with corticosteroids and rituximab. Two patients were given immunosuppressants: 1 with cyclophosphamide and showed improvement, and other was the previously described patient on rituximab who also improved. Overall use of immunosuppressants was higher in SSc group, whereas none of the patients were treated with them specifically for their pseudo-obstruction. Hospitalization was higher in paraneoplastic group, whereas 90-day and 1-year mortality rates were not significantly different between the 3 groups.

**Table 6.**
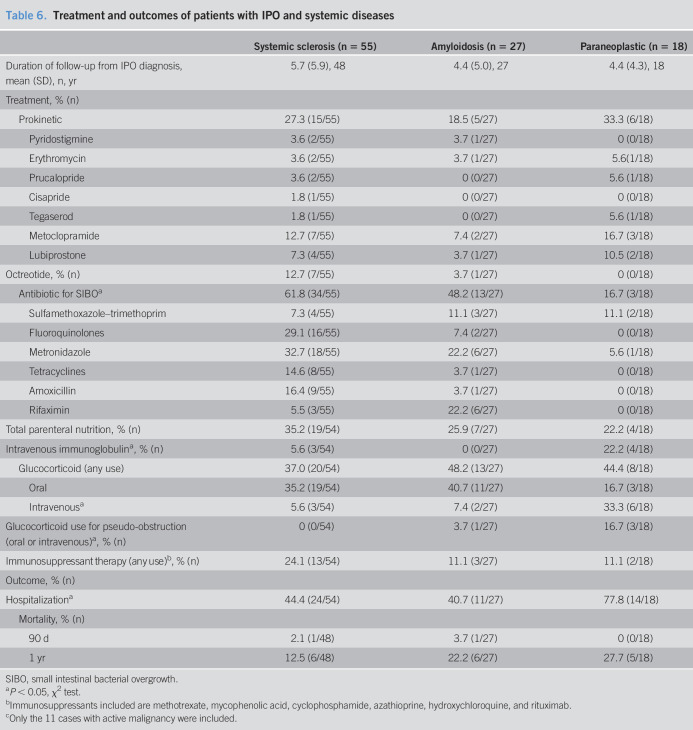
Treatment and outcomes of patients with IPO and systemic diseases

## DISCUSSION

Intestinal pseudo-obstruction, being a rare complication of SSc, amyloidosis, or paraneoplastic syndrome, has only been described in case reports or small case series. To our knowledge, this is the largest single-center series providing detailed clinical, radiological, and physiological characterization of intestinal pseudo-obstruction in these systemic diseases.

Our study reported female population predominance of SSc associated pseudo-obstruction in comparison with that in amyloidosis and paraneoplastic syndrome. No clear effect of sex on GI involvement in SSc was seen in a previous study, except for constipation, which was more common in women (20). Another study also did not find SSc-related GI complications to associate with sex (21). Finally, 1 study showed male sex to be associated with severity of GI dysfunction (22). It is also important to note that the prevalence of SSc is 4- to 5-fold higher among female than male population (23). Age of presentation with SSc-associated pseudo-obstruction was ∼60 years in 2 prior studies (24,25). In a study from Japan, amyloidosis-associated pseudo-obstruction was equally common in male and female population, and age of presentation was 46 years. In our study, 74% were male population, and the mean age at presentation was 60 years (26). The literature on pseudo-obstruction in paraneoplastic syndrome is sparse. The symptom profile of our patients is similar to those reported in previous studies (2,27). Mecoli et al. (24) reported nausea (77%) and abdominal pain (50%) as most common presenting symptoms of pseudo-obstruction, and a study by Schuffler et al. (28) reported nausea and vomiting in all, diarrhea in 96%, and pain in 85% of patients. Lee et al. (19) described paraneoplastic GI dysmotility where the common symptoms were nausea and vomiting (50%) and constipation (17%). In another study of paraneoplastic visceral neuropathy in 7 patients with lung cancer, all patients reported intestinal pseudo-obstruction and constipation (29). In our study, the most common findings were nausea, bloating, and abdominal pain. Diarrhea was more common in SSc and amyloidosis, whereas constipation was more common in paraneoplastic syndrome. Clinically significant weight loss was notable in the SSc group when compared with the other groups (30). A BMI < 18.5 kg/m^2^ is suggestive of protein–energy malnutrition (31,32). Our study showed that a low BMI was more common in patients with SSc than in the patients with paraneoplastic syndromes. The high prevalence of SIBO reported in this and previous SSc studies (33–35) might be the cause of malabsorption and malnutrition. Antibiotic use was also higher in SSc than the other 2 groups, which was expected considering the greater testing for SIBO. Although not significant, total parenteral nutrition use was higher in SSc-associated pseudo-obstruction (35%), compared with the other 2 (22%–26%), which aligns with a high prevalence of malnutrition and weight loss in these patients.

The literature strongly supports that paraneoplastic GI motility is mostly related to SCLC, but in our study, there was only 1 patient with isolated SCLC alone (17,29,36). Three patients presented with squamous cell carcinoma and 1 with both SCLC and squamous cell carcinoma. ANNA-1 was most commonly associated with SCLC. It is believed that ANNA-1 causes activation of the apoptotic cascade resulting in enteric neurodegeneration (18). A study in 12 patients with cancers showed that 8 of 9 patients with SCLC had ANNA-1 positivity, and overall, ANNA-1 was positive in 72.7% of patients (19). In our study, the ANNA-1 antibody was positive in 4 of 18 patients, which might be due to lower numbers of patients with SCLC in our cohort.

One small study of 32 patients with SSc, 27 of which underwent lactulose hydrogen breath test for assessment of orocecal time, showed delayed small bowel transit in 56% patients. Although these patients were otherwise unselected (for GI symptoms or pseudo-obstruction), patients with severe GI dysfunction were excluded from this study (6). In another study, nearly all patients with SSc with pseudo-obstruction had increased small intestinal diameters and only 25% with colonic dilation (28). In our study, small bowel was more commonly involved than large bowel: 79% of patients had only small bowel dilation, 2% had only large bowel dilation, and the remaining had both small and large bowels involved. Duodenal dilation was observed in 96% of patients with a mean diameter of 4.5 cm. Systematic assessment of small and large bowel involvement is not available in amyloidosis and paraneoplastic syndrome. In our study, a fairly similar proportion of patients in these 2 groups had small or large bowel dilation.

An SSc pseudo-obstruction study did not show delay in GE but the small bowel transit time was remarkably delayed (37). However, in another cohort of unselected patients with SSc (n = 14), GE was significantly prolonged in 57% of patients with SSc, delayed orocecal transit in 40% and prolonged whole gut transit in 23% (38). Although GE was delayed in SSc compared with controls (inpatients without GI symptoms or healthy volunteers), small intestinal and whole gut transit times were not significantly different. It is to be noted that hydrogen breath testing and indigo carmine–based assessments of small intestinal and colonic transit were used in this study. Another study of 15 patients with SSc with diffuse abdominal symptoms from Denmark showed prolonged GE in 27% of patients with SSc and an overall GI transit time of >3 days in 53% of patients, both significantly greater than healthy controls (39). Small intestinal contractility was significantly slower in SSc than controls in this study. In our study, GE at 4 hours was delayed in 54% of patients (mean = 71%), small bowel transit was delayed in 68% of patients (mean = 28%), and colonic transit at 24 hours was delayed in 50% of patients. Interestingly, 14%–18% of SSc patients also had accelerated GE which can cause symptoms indistinguishable from delayed GE (40). In a study of small bowel amyloid infiltration, the transit of barium from stomach to colon was prolonged but another study showed rapid transit of chyme in systemic amyloidosis (41,42). Patients presented for assessment of diarrhea in both of these studies. In our study, GE at 4 hours was delayed in 60% of patients, whereas small bowel and colon transit was delayed in all patients. However, only 5 of 27 amyloidosis patients underwent transit studies. A study conducted on mice injected systemically with IgG containing potent ganglionic AChR antibodies showed severe decrease in bowel motility due to disruption of ganglionic cholinergic neurotransmission (43). A retrospective study in patients with lung cancer and pseudo-obstruction showed that 57% had delayed small bowel transit and 43% had delayed colonic transit (29). Another study showed 89% patients with malignant tumors and GI symptoms had delayed GE (19). In our study, 50% patients had abnormal GE and 67% had abnormal small bowel transit. However, colon transit was normal in most patients studied.

Manometry is a key element in the process of disease identification: presence of low-amplitude contractions indicates a myopathic process and uncoordinated contractility indicates a neuropathic process (44). In our analysis, the patients with SSc had low-amplitude contraction denoting a myopathic process, and 60% of patients with paraneoplastic syndromes had uncoordinated contractility denoting a neuropathic process. This could be due to the involvement of myenteric plexus in paraneoplastic syndrome (1). The myopathic type of pseudo-obstruction was reported in the AL subtype with a chronic presentation, whereas the neuropathic type was reported in AA with an acute presentation (45). We did not have manometry performed on most of our patients with amyloidosis, precluding assessment in this subset.

The ability to assess response to treatments in our cohort is limited due to lack of adequate follow-up. Considering we have a tertiary-care practice, a large proportion of our patients follow-up with their local providers for implementing treatment recommendations and assessing response. Regardless, IVIG use was associated with mixed response in both SSc (n = 3) and paraneoplastic (n = 4) groups. None of the patients with amyloidosis were treated with IVIG. Corticosteroids were predominantly used for paraneoplastic pseudo-obstruction, either alone or in combination with other immunosuppressants. In most patients, their use was associated with a favorable response. Outside of isolated case reports, not much data are available on treatment of paraneoplastic intestinal pseudo-obstruction, and our study provides the largest number of cases with available treatment response.

Our study uses the strength of having detailed clinical and physiological characterization available on these patients from a single tertiary-care center with expertise in evaluation of motility disorders. Importantly, comprehensive imaging and motility findings have not been described in previous cohorts. Our sample size was large enough to drive conclusions on similarities and differences between pseudo-obstructions associated with these disorders. The limitations include the retrospective and descriptive nature of the case series and lack of controls. Our study required thresholds for bowel dilation on imaging to be included as pseudo-obstruction. This would likely influence the demographic and clinical features we observed vs those reported in other studies. The small size of the paraneoplastic group in our study with limited number of associated malignancies and onconeural antibodies also makes it difficult to compare the results of our study with those of other studies of paraneoplastic dysmotility. Moreover, not all patients underwent manometric studies that can be invasive and do not always guide changes in management.

In conclusions, SSc-associated pseudo-obstruction is more commonly associated with vomiting, diarrhea, and weight loss. The small bowel is more commonly involved than the large bowel on both imaging and transit studies in patients with SSc, whereas both small and large bowel showed equal involvement in the other 2 groups. Myopathic involvement was more common in SSc, whereas neuropathic involvement was more common in paraneoplastic syndrome, but this warrants further study in a larger cohort of patients. Intestinal pseudo-obstruction should be recognized as an important complication with these systemic diseases and addressed to alleviate complications such as malnutrition.

## CONFLICTS OF INTEREST

**Guarantor of the article:** Madhusudan Grover, MD and Ashima Makol, MD.

**Specific author contributions:** R.P.: analysis and interpretation of data; drafting of the manuscript; and critical revision of the manuscript. A.B.: analysis and interpretation of data; drafting of the manuscript; and critical revision of the manuscript. M.B.-L.: analysis and interpretation of data and critical revision of the manuscript. M.G.: study concept and design; analysis and interpretation of data; drafting of the manuscript; critical revision of the manuscript; and study supervision. A.M.: study concept and design, analysis and interpretation of data; critical revision of the manuscript; and study supervision.

**Financial support:** This research was supported by the funds from Division of Gastroenterology and Hepatology at Mayo Clinic. In addition, Dr Grover is supported by NIDDK K23 103911 and R03 120745.

**Potential competing interests:** None to report.

**Study Highlights**

**WHAT IS KNOWN**

✓ Intestinal pseudo-obstruction is a rare, disabling, and poorly understood complication of systemic diseases.
✓ SSc, amyloidosis, and paraneoplastic syndrome have been described to cause intestinal pseudo-obstruction, but their clinical correlates are poorly defined.

**WHAT IS NEW HERE**

✓ SSc-associated intestinal pseudo-obstruction demonstrates female population predominance in comparison with amyloidosis-associated pseudo-obstruction that demonstrates male population predominance.
✓ SSc-associated intestinal pseudo-obstruction predominantly affects the small bowel and causes vomiting, diarrhea, and significant weight loss, whereas amyloidosis and paraneoplastic syndrome involve both small and large bowel.
✓ Myopathic involvement is more common in SSc, whereas paraneoplastic syndrome–related pseudo-obstruction is neuropathic in nature.

**TRANSLATIONAL IMPACT**

✓ Recognition and assessment of symptoms of intestinal pseudo-obstruction is important in patients with SSc, amylodosis and paraneoplastic syndrome which may result in significant malnutrition and contribute to the overall burden associated with these disorders.
